# Manual Physical Therapists' Use of Biopsychosocial History Taking in the Management of Patients with Back or Neck Pain in Clinical Practice

**DOI:** 10.1155/2015/170463

**Published:** 2015-04-05

**Authors:** Rob A. B. Oostendorp, Hans Elvers, Emilia Mikołajewska, Marjan Laekeman, Emiel van Trijffel, Han Samwel, William Duquet

**Affiliations:** ^1^Department of Manual Therapy, Faculty of Medicine and Pharmacy, Vrije Universiteit Brussel, Campus Jette, Laarbeeklaan 103, 1050 Brussels, Belgium; ^2^Scientific Institute for Quality of Healthcare, Radboud University Nijmegen Medical Centre, P.O. Box 9100, 6500 HB Nijmegen, Netherlands; ^3^Faculty of Physical Education and Physiotherapy, Pain in Motion Research Group, Campus Etterbeek, Pleinlaan 2, 1050 Brussels, Belgium; ^4^Department of Public Health and Research, Radboud University Nijmegen Medical Centre, P.O. Box 9100, 6500 HB Nijmegen, Netherlands; ^5^Institute for Methodology and Statistics Beuningen, Hofstee 10, 6641 VP Beuningen, Netherlands; ^6^Rehabilitation Clinic, Clinical Military Hospital No. 10 with Polyclinic, Powstańców Warszawy 5, 85-681 Bydgoszcz, Poland; ^7^Neurocognitive Laboratory, Centre for Modern Interdisciplinary Technologies, Nicolaus Copernicus University, Wileńska 4, 87-100 Toruń, Poland; ^8^Department of Nursing Sciences, Faculty of Health, Witten/Herdecke University, Stockumer Straße 12, 58453 Witten, Germany; ^9^SOMT Educational Institute for Musculoskeletal Therapy, Softwareweg 5, 3821 BN Amersfoort, Netherlands; ^10^Department of Medical Psychology, Radboud University Nijmegen Medical Centre, P.O. Box 9100, 6500 HB Nijmegen, Netherlands; ^11^Department of Statistics, Faculty of Physical Education and Physiotherapy, Vrije Universiteit Brussel, Campus Oefenplein, Pleinlaan 2, 1050 Brussels, Belgium

## Abstract

*Objective.* To develop and evaluate process indicators relevant to biopsychosocial history taking in patients with chronic back and neck pain. *Methods.* The SCEBS method, covering the Somatic, Psychological (Cognition, Emotion, and Behavior), and Social dimensions of chronic pain, was used to evaluate biopsychosocial history taking by manual physical therapists (MPTs). In Phase I, process indicators were developed while in Phase II indicators were tested in practice. *Results.* Literature-based recommendations were transformed into 51 process indicators. Twenty MTPs contributed 108 patient audio recordings. History taking was excellent (98.3%) for the Somatic dimension, very inadequate for Cognition (43.1%) and Behavior (38.3%), weak (27.8%) for Emotion, and low (18.2%) for the Social dimension. MTPs estimated their coverage of the Somatic dimension as excellent (100%), as adequate for Cognition, Emotion, and Behavior (60.1%), and as very inadequate for the Social dimension (39.8%). *Conclusion.* MTPs perform screening for musculoskeletal pain mainly through the use of somatic dimension of (chronic) pain. Psychological and social dimensions of chronic pain were inadequately covered by MPTs. Furthermore, a substantial discrepancy between actual and self-estimated use of biopsychosocial history taking was noted. We strongly recommend full implementation of the SCEBS method in educational programs in manual physical therapy.

## 1. Introduction

Since the introduction of the biopsychosocial disease model by Engel [1], there has been a considerable shift in the use of this model for the diagnosis and management of musculoskeletal disorders, such as back and neck pain. In the past, the biomedical model predominantly focused on anatomical structures related to the back and neck region as the origin of pain and as justification for medical interventions. The subsequent failure of many treatment approaches, amongst other factors, highlighted the limitations of the biomedical model in the treatment of patients with musculoskeletal disorders.

Together with contributions from many other similar papers, a publication by Waddell in 1987 in particular catalyzed the worldwide introduction of the biopsychosocial model for patients with spinal disorders [2]. The last 40 years have seen a surge in research on neuro- and behavioral sciences, including those related to the field of manual physical therapy [3–5]. This has led to a greater appreciation of the role of psychological and social factors that impact (chronic) musculoskeletal pain. A number of factors, including the high incidence and prevalence of patients with (chronic) musculoskeletal pain, the accumulating evidence supporting a role for psychological and social factors in relation to chronic pain, the increasing number of clinical practice guidelines based on scientific evidence, the international classifications (e.g., International Classification of Functioning, Disability and Health (ICF)) [6], and the growing interest in the clinical reasoning process, point to the relevance of a broader approach to the management of patients with musculoskeletal disorders. Despite this, little is still known about the extent to which manual physical therapists (MTPs) apply the biopsychosocial concept in their process of clinical reasoning for patients with musculoskeletal pain, particularly nonspecific back and neck pain.

The process of clinical reasoning consists of a diagnostic phase (history taking, [objectives of] physical examination, analysis, and conclusion), a treatment phase (treatment plan and treatment), and an evaluation phase (evaluation and discharge). The “history taking” is the first step in the diagnostic phase and is crucial to the orientation on the health problem of patients with (chronic) musculoskeletal pain in terms of (impairments in) body functions and structures, activity (limitations), participation (restrictions), and personal and environmental factors. The SCEBS method, developed in 1995 by Van Spaendonck and Bleijenberg (medical psychologists), is designed as a diagnostic frame work for general practitioners who are less familiar with the biopsychosocial history taking in patients with (chronic) pain. [7–9]. This method identifies three dimensions of pain: the Somatic or biological dimension, the Psychological dimension (Cognition, Emotion, and Behavior), and the Social dimension. A set of sample questions was developed for each dimension such as “Can you move your back/neck?” (to trace impairments of movement-related functions), “What do you think when you are experiencing pain?” (to trace catastrophic or helplessness cognitions, fear of pain, lack of self-efficacy, or unrealistic treatment expectations), “How do you feel when you experience pain?” (to trace depression or anxiety), “What do you in response of pain?” (to trace avoidance behavior or pain resistance behavior), and “How does your social environment react to your pain?” (to trace maladaptive social responses to pain behavior). The SCEBS method is commonly used in the Netherlands by general practitioners, occupational physicians, psychologists, nurses, and to a lesser extent MTPs, for the initial orientation and analysis in patients with inexplicable and unexplained pain [10, 11].

The transparency of the SCEBS method-based process of history taking, using measurable elements such as Quality Indicators (QIs), is seen as one of the cornerstones of the quality of care, particularly the quality of manual physical therapy in patients with (chronic) musculoskeletal pain. QIs have been defined as “measurable elements of practice performance for which there is evidence or consensus that they can be used to assess the quality, and thus change the quality, of care provided” [12, 13]. QIs are related to structures (such as staff, equipment, and appointment systems), processes (such as prescribing, investigations, and clinical reasoning), and outcomes (such as mortality, morbidity, patient satisfaction, and functioning) of care [14]. QIs are preferably derived from guideline-based recommendations, supplemented by expert clinical experience and patient perspectives and developed by means of a systematic method [15]. After development, sets of QIs should be subjected to a pilot practice test.

The present study focused on the development and evaluation of process indicators in patients with chronic musculoskeletal pain, with an emphasis on nonspecific back and neck pain. The two primary goals of this study were (1) to develop a set of process indicators relevant to biopsychosocial history taking in patients with nonspecific back and neck pain and (2) to subject this set to a pilot practice test to determine its value in assessing the actual extent of implementation of biopsychosocial history taking in Dutch manual physical therapy practice.

## 2. Materials and Methods

### 2.1. Design

The study consisted of two phases: (i) indicator development and (ii) indicator testing through a pilot practice test. The QI development included three steps: (i) extraction of recommendations from original description of the SCEBS method and related literature, (ii) transformation of recommendations into process indicators, and (iii) classification of process indicators according to the SCEBS method. For the pilot practice test, we used an observational prospective cross-sectional design to test the integration of biopsychosocial history taking in manual physical therapy practice.

The Medical Ethics Committee of Radboud University Medical Centre Nijmegen in the Netherlands stated in writing that ethical approval was not necessary. Each practice formally consented to participate in the study and all patients were informed about the study and gave permission for anonymous use of data.

### 2.2. Phase  1: Indicator Development

#### 2.2.1. Step  1: Extraction of Recommendations

Recommendations were identified using the original SCEBS method literature, systematic reviews of the screening, assessment and management of patients with nonspecific back or neck pain, and core ICF sets for musculoskeletal disorders. These recommendations were extracted by two members of the research team (Rob A. B. Oostendorp and William Duquet) and, where necessary, differences were discussed with a third member (Peter Vaes) until consensus was reached. Based on these recommendations, a set of questions was formulated for each dimension (e.g., the Somatic dimension: what are the type, localization, intensity, frequency, and duration of pain, and what are the impairments of neuromusculoskeletal and movement-related functions (such as mobility and stability of joint functions); Cognition: what are your expectations of treatment?).

#### 2.2.2. Step  2: Transformation into Process Indicators

The questions were transformed into process indicators by treating them as percentages of patients who were asked a certain question (i.e., the percentage of patients who were asked specific questions about their attributions of pain).

#### 2.2.3. Step  3: Classification of Process Indicators

The process indicators were classified into the three dimensions of the SCEBS method with eight subdimensions of the Psychological dimension (appendix).

### 2.3. Phase  2: Indicator Testing

**Figure 1:**
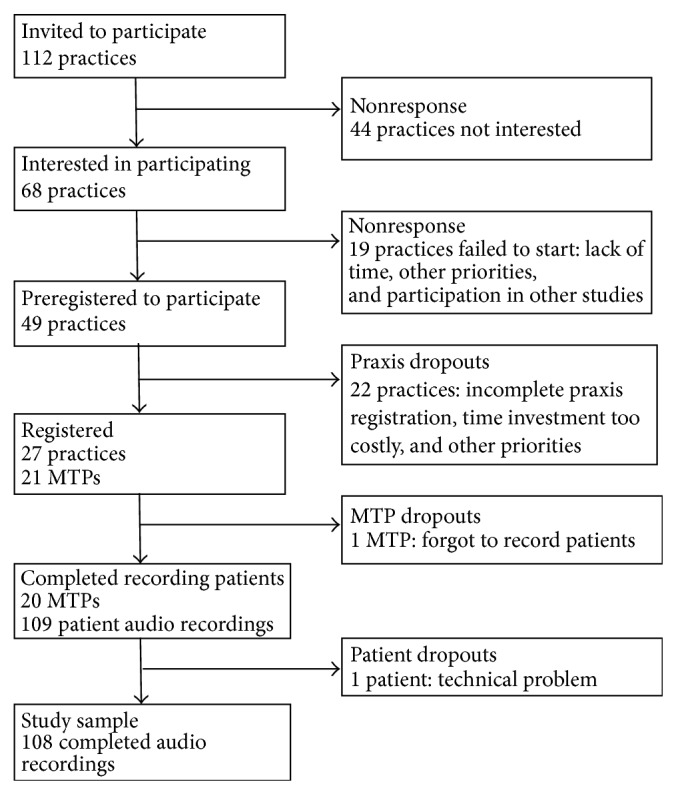
Flowchart of manual physical therapy practices and participant manual physical therapists' (MTPs') responses and reasons for nonresponse and dropouts.

An invitation to participate in the pilot practice test was sent to 112 physiotherapy practices in the south of the Netherlands, of which 68 (60.7%) indicated interest (Figure 1).

From the 68 practices, MTPs from 49 practices (72.1%) participated in a regional information session that outlined the purpose and content of the study and the expected contribution. Of the 49 practices, 27 (55.1%) enrolled 21 MTPs. These MTPs were asked to collect data on at least five new patients with nonspecific back or neck pain, preferably on the first new patient each week meeting the criteria. Based on the number of participating MTPs, the number of patients expected to be included in the study was about 100. Patients had to meet the following inclusion criteria: age from 18 to 70 years, pain and/or stiffness in the back or neck for at least six weeks, back or neck complaints reproducible during active or passive examination, and written informed consent. Nonspecific back or neck pain was defined as pain with no specific cause, such as systemic disease, fracture, or other organic disorder. Patients with a history of additional complaints, such as nonradicular pain, were included only if the back or neck pain was dominant. Patients whose history, signs, and symptoms suggested a potential nonbenign cause (including previous surgery of the back or neck) or who showed evidence of a specific pathologic condition, such as malignancy, neurologic disease, fracture, herniated disc, or systemic rheumatic disease, were excluded.

### 2.4. Data Collection

Data were collected over a period of six months. The history taking during the first appointment took place in the MTP's practice and was recorded using digital audio recording equipment. The audio recordings were transcribed by four students supervised and checked by Rob A. B. Oostendorp and William Duquet. The questions posed by the MTPs and the patients' answers were counted. The patients' demographic and clinical characteristics were also recorded. The age, gender, clinical experience, and additional educational attainment of the MTPs were noted. To evaluate the extent of self-estimated use of biopsychosocial history taking, the MTPs were subsequently asked if all dimensions of the SCEBS method were dealt with.

### 2.5. Data Analysis

The transcripts were read several times by each of the students and supervisors, in order to achieve familiarity with the contents of the questions and answers during history taking. Significant phrases were identified that characterized a specific question and answer of a (sub)dimension of the SCEBS method of history taking. One point was scored for each question that adhered to (sub)dimensions of the SCEBS method.

Process indicators were scored as percentages (yielding possible scores for the use of biopsychosocial history taking ranging from 0 to 100%), with the number of times an indicator was met as the numerator and the number of patients assessed as the denominator. To allow for easy interpretation, percentage scores of process indicators were categorized as negligible (0–15%), low (16–25%), weak (26–35%), very inadequate (36–45%), inadequate (46–55%), adequate (56–65%), substantial (66–75%), good (76–85%), very good (86–95%), and excellent (96–100%). The cut-off point for acceptable coverage for every dimension was set at 60%.

The estimated extent of the use of biopsychosocial history taking by the MTPs themselves was expressed as percentages using the same categorization as above.

## 3. Results

### 3.1. Phase  1: Indicator Development

Sixty-eight literature-based recommendations were extracted for biopsychosocial history taking in patients with nonspecific back or neck pain. After critical evaluation and checking for duplication and overlap by two members of the project group (Rob A. B. Oostendorp and William Duquet), the number of preselected recommendations was reduced to 51 items.

The recommendations were transformed into 51 process indicators: for instance, “the percentage of patients who were asked about their own influence on their complaints,” “the percentage of patients who were asked about the reaction of their social environment to their complaints,” or “the percentage of patients who were asked about fear related to certain physical activities” (see appendix).

The process indicators were classified into the dimensions of the SCEGS method: Somatic dimension (*n* = 10), Psychological dimension (Cognition *n* = 14; Emotion *n* = 6; Behavior *n* = 11), and Social dimension (*n* = 10).

### 3.2. Phase  2: Indicator Testing

#### 3.2.1. Response Rates

Of the 21 registered MTPs, 20 (95.2%) submitted data to the pilot practice test (Figure 1). One hundred and nine patients participated in the study, of whom one was excluded from the analysis due to a technical problem with the audio recording, leaving 108 patient recordings in the study.

#### 3.2.2. Participating Manual Physical Therapists and Patients

The mean age of the MTPs (*n* = 20) was 40.7 years (SD = 8.5), of whom 45.0% (*n* = 9) were female. All participants had postgraduate level education in manual therapy (Stichting Opleiding Manuele Therapie (SOMT) (Educational Institute for Manual Therapy), Amersfoort, Netherlands). The range of practice experience was eight to 22 years. The average age of the patients (*n* = 108) was 42.3 years (SD = 14.1) of whom 60 (55.6%) were female. Of the 108 patients, 68 (62.9%) had back pain and 40 (37.0%) had neck pain.

#### 3.2.3. Use of Biopsychosocial History Taking

Average percentage scores for the use of biopsychosocial history taking according to the QIs classified into the dimensions of the SCEBS method (Table 1) indicated that the extent to which the participating MTPs met the process indicators was excellent for the Somatic dimension (98.1%), very inadequate for Cognition (42.5%) and Behavior (37.9%), weak for Emotion (26.8%), and low for the Social dimension (17.6%). The coverage of the Somatic dimension was above the cut-off criterion of 60%.

Average percentage scores for the self-estimated extent of use of biopsychosocial history taking according to the SCEBS method by the MTPs themselves (Table 1) indicated that the level of use of the Somatic dimension was excellent (100%), adequate for Cognition, Emotion, and Behavior (60.1%) of the Psychological dimension, and very inadequate for the Social dimension (39.8%).

## 4. Discussion

This study demonstrates that the use in clinical practice of manual physical therapy of biopsychosocial history taking in patients with back or neck pain varies widely across the various dimensions of the SCEBS method. In particular, the Psychological and Social dimensions of (chronic) pain were inadequately covered during history taking in these patients. Although we could not find a comparable study in the literature, these data are consistent with studies of physical therapy care that showed poorer quality in the implementation of biopsychosocial management of musculoskeletal disorders than in the implementation of biomedical management for back and neck pain [16]. These results suggest that MTPs involved in the primary care of patients with (chronic) musculoskeletal disorders need more in-depth training in biopsychosocial history taking, preferably adopting the SCEBS method, along with continuing education to develop and maintain skills [17, 18]. With the notable exception of the Somatic dimension, it is striking that the participating MTPs overestimated their use of biopsychosocial history taking. It is possible that during patient contacts biopsychosocial information is added and subsequently integrated into the clinical reasoning processes [17–21] and a prospective study with follow-up of patient contacts could reveal the subsequent gathering of such information.

MTPs should be familiar not only with biopsychosocial context of pain but also with modern insights from pain neuroscience concerning reconceptualization of pain [22]. A sustained biomedical approach can lead to an iatrogenic effect which results in an increase in pain [23]. Although there is increasing evidence supporting the role of psychological and social factors in the emergence and persistence of chronic musculoskeletal pain, the majority of clinicians received a biomedically focused education, a focus that is also evident in the profession of manual physical therapy. This focus is reflected in a long tradition of treatment options based on biomechanical principles in patients with musculoskeletal disorders. This emphasis on biomedical aspects likely shapes therapists' knowledge, attitudes, beliefs, and behavior towards (chronic) musculoskeletal pain [22, 23]. In addition, the emergence of new or revised theory and subsequent changes in practice are often characterized by a significant time-lag. The integration of the biopsychosocial model into daily practice is therefore challenging, especially for those practitioners who did not receive formal education in the application in this model in clinical assessments. The concept that (chronic) musculoskeletal pain is a condition best understood with reference to an interaction of physical (biological), psychological, and social factors is increasingly accepted in manual physical therapy. It is therefore not surprising that this acceptance has led to discussion of the value of manual physical therapy as a one-dimensional (physical) assessment in patients with back or neck pain. This has resulted in the integration of psychological and social factors in clinical practice guidelines and in multidisciplinary biopsychosocial rehabilitation programs [24]. It has also been suggested that multimodal treatments are superior to unimodal treatment (e.g., manual physical therapy).

Despite the development of many (theoretical) implementation strategies and activities in the field of manual physical therapy [25, 26], programs to enhance guideline adherence (including the use of standardized measurement instruments or questionnaires) have so far been relatively ineffective [27–31]. It has been reported that MTPs exhibit only moderate adherence to clinical practice guidelines, and research carried out in the Netherlands has revealed that a lack of knowledge and competencies of physical therapists with respect to the use of measurement instruments and questionnaires may hamper the implementation of guidelines [32].

This study describes the development of QIs to measure the use of biopsychosocial history taking as a first step in clinical practice guidelines associated with (chronic) musculoskeletal pain in patients with back or neck pain. Additional evidence indicates that many interfering factors in relation to pain can only be identified by careful history taking [33]. The SCEBS method is the most commonly used method in the Netherlands for a systematic inventory and analysis of factors related to pain and this method is also integrated in the revision and actualization of clinical practice guidelines by the Royal Dutch Society for Physical Therapy (e.g., Low Back Pain [34]).

Although no formal external validation of the set of QIs has taken place, the systematic approach and the composition of the research group underline the content validity of the QIs set derived from the SCEBS method. External validity depends on the heterogeneity of the expert panel, which consists of patient representatives, psychologists, general physicians, MTPs, and teachers. There is a pressing need for further research in the aforementioned area that includes larger groups of both experts and patients. While in this case the response rate of participating practices was acceptable (55.1%), the relatively small self-selected sample of participating MTPs might limit the external validity of the practice test. The MTPs were comparable to national profiles for this group [35] and patients were comparable to participants in other Dutch studies [27, 36–38]. With a target of five patients per participant, the number of patients was adequate. The high patient response was probably due to the limited burden of recording the history taking with an audio recorder, in contrast to the greater time commitments of a randomized study or the repeated filling of questionnaires for the evaluation of treatment in clinical practice [32]. Lack of time is one of the reasons for not entering or no longer participating in clinical studies.

In addition to the years of clinical experience, the majority of participating MTPs were also educated in the biomedical model of pain. Unsurprisingly, the use of Somatic history taking was “excellent” in this study. By contrast, the use of the Psychological and Social dimension was “very inadequate” to “low.” Unlike recent graduate MTPs, it might have been expected that a group of MTPs with long clinical experience would have integrated the biopsychosocial approach into their first contacts with patients. In a qualitative study, Agledahl et al. [39] found that young doctors or doctors in training largely ignore the impact of symptoms on patient's daily life. This biomedical approach suggests that the next steps in the clinical reasoning process will be defined by the results of the preceding biomedical history taking. Manual physical therapy is often presented as a treatment option to patients with back or neck pain within this biomedical model of pain. Traditionally, the objectives of manual physical therapy have been to find impairments in body functions and structures related to posture and movement, which MTPs then treat using hands-on techniques (e.g., mobilization or manipulation of joints). In this pilot study, only data on history taking are available; no data on the remaining steps of the diagnostic and therapeutic process and the outcome of the treatment were gathered. This may be regarded as a limitation of the study.

A large number of published studies and (systematic) review articles in various journals (e.g., Pain and Manual Therapy) advocate a broader view of (chronic) musculoskeletal pain [3, 5, 17, 20, 40–42]. Based on this literature and the results of this study, we urge to make this broader vision their own. An increasing number of manual physical therapy curricula around the world now emphasize the biopsychosocial model in their educational programs and teach communication skills in addition to hands-on techniques [43, 44]. Recent research clearly demonstrates that musculoskeletal pain is a heterogeneous condition involving biological, psychological, and social factors to varying degrees. Biopsychosocial history taking, using a method such as SCEBS, in combination with the ICF and modern insights from pain neuroscience, plays a central role in the inventory of biological, psychological, and social factors and consequently in the next steps of the clinical reasoning process of MTPs.

## 5. Conclusion

In summary, our results indicate that MTPs perform screening for musculoskeletal pain mainly through the use of the Somatic or biomedical dimension of (chronic) pain according to the SCEBS method in patients with back or neck pain. Psychological and Social dimensions of chronic pain were inadequately covered by MPTs. There is a substantial discrepancy between the actual and self-estimated use of biopsychosocial history taking. Further work should focus on the role of education of MTPs in promoting complete biopsychosocial history taking and follow-through within the diagnostic, therapeutic, and evaluative phases of the clinical reasoning process.

## Figures and Tables

**Table 1:** Use of biopsychosocial history taking according to the SCEBS method: number of quality indicators (appendix) and number and percentage scores for actual and self-estimated use by manual physical therapists (*n* = 20) in patients with back or neck pain (*n* = 108).

History taking	Actual use	Self-estimated use
	*n* (%)	*n* (%)
S = Somatic dimension		
10 indicators	106 (98.1)	108 (100)
Psychological dimension		
C = Cognition		
14 indicators	46 (42.5)	65 (60.1)
E = Emotion		
6 indicators	29 (26.8)	65 (60.1)
B = Behavior		
11 indicators	41 (37.9)	65 (60.1)
S = Social dimension		
10 indicators	19 (17.6)	43 (39.8)

## Appendix

### A. SCEBS Method (Dutch: SCEGS Methode)

#### A.1. S = Somatic Dimension (Dutch: Somatische Dimensie)

1. What are your complaints?
2. When did the complaints begin?
3. What are the nature, the location, and the intensity of the complaints?
4. How often do the symptoms occur?
5. How long do the symptoms last?
6. Have you had these symptoms before?
7. Can you move your back/neck?
8. Have you experienced any stiffness?
9. What do the X-ray results show?
10. What do laboratory tests show?

#### A.2. Psychological Dimension (Dutch: Psychologische Dimensie)

##### A.2.1. C = Cognition (Dutch: Cognitie)

*Expectations*

(11) What do you expect from me?
(12) What do you think I can do for you?

*Explanations (Attribution)*

(13) What do you think yourself?
(14) Do you yourself have any explanation for your complaints?
(15) Do you sometimes think “if it isn't this or that”?

*Thinking about Complaints/Thinking That Worsens Complaints (Catastrophizing)*

(16) How do you feel when you have symptoms?
(17) What do you think at that moment?
(18) How do you react?

*Ideas about Personal Influence on Complaints (Self-Efficacy)*

(19) Do you personally have any influence on the complaint?
(20) Can you positively influence the complaint?
(21) If so, how?
(22) Is there anything you yourself can do to reduce your complaint?
(23) Do complaints resolve more quickly when you rest?
(24) Do complaints lessen when you think about something or someone else?

##### A.2.2. E = Emotion (Dutch: Emotie)

(25) Given that you have these complaints, how do you feel about it?
(26) Do the complaints disturb your emotional balance?
(27) Are you insecure?
(28) Are you depressed?
(29) Are you anxious?
(30) Do you ever feel overwhelmed by the complaints?

##### A.2.3. B = Behavior (Dutch: Gedrag)

*Dealing with the Complaint*

(31) What do you do if you have symptoms?
(32) What do you do to reduce symptoms?
(33) To what extent is this successful?

*Limitations to Activities*

(34) Which activities are hindered by your complaints?
(35) To what extent?

*Avoidance*

(36) What do not you do or no longer do when you have symptoms?
(37) Since when?
(38) Are you anxious about particular activities?
(39) What do other people notice about your behavior when you have symptoms?

*Talking about Complaints*

(40) Do you talk about your complaints? With whom? How often?
(41) What do you tell them?

#### A.3. S = Social Dimension (Dutch: Sociale Dimensie)

(42) Do the people around you notice when you have complaints?
(43) What do they notice?
(44) How do you react to your complaints?
(45) What do the people around you think about your complaints?
(46) How do the people around you react to your complaints?
(47) Where does your partner think that your complaints come from?
(48) How did the people around you react when you told them what the doctor said?
(49) How do you now feel about this?
(50) Do the complaints affect your social life?
(51) Did you need to adapt your work/hobby/sport to your complaints?

## References

[1] Engel G. L. (1978). The biopsychosocial model and the education of health professionals. *Annals of the New York Academy of Sciences*.

[2] Waddell G. (1987). A new clinical model for the treatment of low-back pain. *Spine*.

[3] Nijs J., Torres-Cueco R., van Wilgen C. P. (2014). Applying modern pain neuroscience in clinical practice: criteria for the classification of central sensitization pain. *Pain Physician*.

[4] Roussel N. A., Nijs J., Meeus M., Mylius V., Fayt C., Oostendorp R. (2013). Central sensitization and altered central pain processing in chronic low back pain: fact or myth?. *Clinical Journal of Pain*.

[5] Vlaeyen J. W. S., Linton S. J. (2012). Fear-avoidance model of chronic musculoskeletal pain: 12 years on. *Pain*.

[6] WHO (2001). *International Classification of Functioning, Disability and Health*.

[7] Van Spaendonck K. P. M. *Functionele Klachten in de Medische Praktijk: een Werkmodel*.

[8] Van Duppen D., Neirincks J., Seuntjens L. (2005). Van counselen naar cognitieve gedragstherapie. *Huisarts Nu*.

[9] van Spaendonck K. P. M., Bleijenberg G. http://www.wikifysio.nl/index.php/Biopsychosociale_klachten,_SCEGS.

[10] Olde Hartman T. C., Blankenstein A. H., Molenaar A. O. (2013). NHG-Standaard Somatisch Onvoldoende verklaarde Lichamelijk Klachten (SOLK). *Huisarts en Wetenschap*.

[11] Hoedeman R., Wijers J. H. L., van der Beek E. J., Te Koppele A. (2006). Toepassing van het SCEGS-model in de begeleiding van somatiserende werknemers. *Tijdschrift voor Bedrijfs- en Verzekeringsgeneeskunde*.

[12] Lawrence M., Olesen F. (1997). Indicators of quality in health care. *European Journal of General Practice*.

[13] Grol R., Wensing M., Eccles M. (2005). *Improving Patient Care. The Implementation of Change in Clinical Practice*.

[14] Mainz J. (2003). Defining and classifying clinical indicators for quality improvement. *International Journal for Quality in Health Care*.

[15] Guyatt G. H., Oxman A. D., Kunz R. (2008). GRADE: going from evidence to recommendations. *British Medical Journal*.

[16] Green A. J., Jackson D. A., Klaber Moffett J. A. (2008). An observational study of physiotherapists' use of cognitive-behavioural principles in the management of patients with back pain and neck pain. *Physiotherapy*.

[17] Nijs J., Roussel N., van Wilgen C. P., Köke A., Smeets R. (2013). Thinking beyond muscles and joints: therapists' and patients' attitudes and beliefs regarding chronic musculoskeletal pain are key to applying effective treatment. *Manual Therapy*.

[18] Nijs J., Van Houdenhove B., Oostendorp R. A. B. (2010). Recognition of central sensitization in patients with musculoskeletal pain: application of pain neurophysiology in manual therapy practice. *Manual Therapy*.

[19] Domenech J., Sánchez-Zuriaga D., Segura-Ortí E., Espejo-Tort B., Lisón J. F. (2011). Impact of biomedical and biopsychosocial training sessions on the attitudes, beliefs, and recommendations of health care providers about low back pain: a randomized clinical trial. *Pain*.

[20] van Wilgen P., Beetsma A., Neels H., Roussel N., Nijs J. (2014). Physical therapists should integrate illness perceptions in their assessment in patients with chronic musculoskeletal pain; a qualitative analysis. *Manual Therapy*.

[21] Van Trijffel E., Plochg T., van Hartingsveld F., Lucas C., Oostendorp R. A. B. (2010). The role and position of passive intervertebral motion assessment within clinical reasoning and decision-making in manual physical therapy: a qualitative interview study. *Journal of Manual and Manipulative Therapy*.

[22] Ostelo R. W. J. G., Vlaeyen J. W. S. (2008). Attitudes and beliefs of health care providers: extending the fear-avoidance model. *Pain*.

[23] Darlow B., Fullen B. M., Dean S., Hurley D. A., Baxter G. D., Dowell A. (2012). The association between health care professional attitudes and beliefs and the attitudes and beliefs, clinical management, and outcomes of patients with low back pain: a systematic review. *European Journal of Pain*.

[24] Kamper S. J., Apeldoorn A. T., Chiarotto A. (2014). Multidisciplinary biopsychosocial rehabilitation for chronic low back pain. *Cochrane Database of Systematic Reviews*.

[25] Jones M., Edwards I., Gifford L. (2002). Conceptual models for implementing biopsychosocial theory in clinical practice. *Manual Therapy*.

[26] Bekkering G. E., Engers A. J., Wensing M. (2003). Development of an implementation strategy for physiotherapy guidelines on low back pain. *Australian Journal of Physiotherapy*.

[27] Bekkering G. E., Hendriks H. J. M., van Tulder M. W. (2005). Effect on the process of care of an active strategy to implement clinical guidelines on physiotherapy for low back pain: a cluster randomised controlled trial. *Quality and Safety in Health Care*.

[28] Rutten G. M., Degen S., Hendriks E. J., Braspenning J. C., Harting J., Oostendorp R. A. (2010). Adherence to clinical practice guidelines for low back pain in physical therapy: do patients benefit?. *Physical Therapy*.

[29] Swinkels I. C. S., van den Ende C. H. M., van den Bosch W., Dekker J., Wimmers R. H. (2005). Physiotherapy management of low back pain: does practice match the Dutch guidelines?. *Australian Journal of Physiotherapy*.

[30] Oostendorp R. A. B., Rutten G. M., Dommerholt J., Nijhuis-van der Sanden M. W., Harting J. (2013). Guideline-based development and practice test of quality indicators for physiotherapy care in patients with neck pain. *Journal of Evaluation in Clinical Practice*.

[31] van Dulmen S. A., Maas M., Staal J. B. (2014). Effectiveness of peer assessment for implementing a Dutch physical
therapy low back pain guideline: cluster randomized controlled
trial. *Physical Therapy*.

[32] Swinkels R. A. H. M., van Peppen R. P. S., Wittink H., Custers J. W. H., Beurskens A. J. H. M. (2011). Current use and barriers and facilitators for implementation of standardised measures in physical therapy in the Netherlands. *BMC Musculoskeletal Disorders*.

[33] Chodosh J., Ferrell B. A., Shekelle P. G., Wenger N. S. (2001). Quality indicators for pain management in vulnerable elders. *Annals of Internal Medicine*.

[34] Staal J. B., Hendriks E. J. M., Heijmans M. *KNGF-Richtlijn Lage Rugpijn*.

[35] Kenens R., Hingstman L. (2008). *Cijfers uit de registratie van fysiotherapeuten*.

[36] Picavet H. S. J., Schouten J. S. A. G. (2003). Musculoskeletal pain in the Netherlands: prevalences, consequences and risk groups, the DMC3-study. *Pain*.

[37] Pool J. J. M., Ostelo R. W., Knol D. L., Vlaeyen J. W. S., Bouter L. M., de Vet H. C. W. (2010). Is a behavioral graded activity program more effective than manual therapy in patients with subacute neck pain? Results of a randomized clinical trial. *Spine*.

[38] Hoving J. L., de Vet H. C. W., Koes B. W. (2006). Manual therapy, physical therapy, or continued care by the general practitioner for patients with neck pain: long-term results from a pragmatic randomized clinical trial. *Clinical Journal of Pain*.

[39] Agledahl K. M., Gulbrandsen P., Førde R., Wifstad Å. (2011). Courteous but not curious: how doctors' politeness masks their existential neglect. A qualitative study of video-recorded patient consultations. *Journal of Medical Ethics*.

[40] Nijs J., Meeus M., Cagnie B. (2014). A modern neuroscience approach to chronic spinal pain: combining pain neuroscience education with cognition-targeted motor control training. *Physical Therapy*.

[41] Nijs J., van Wilgen C. P., van Oosterwijck J., van Ittersum M., Meeus M. (2011). How to explain central sensitization to patients with “unexplained” chronic musculoskeletal pain: practice guidelines. *Manual Therapy*.

[42] Meeus M., Nijs J., van Oosterwijck J., van Alsenoy V., Truijen S. (2010). Pain physiology education improves pain beliefs in patients with chronic fatigue syndrome compared with pacing and self-management education: a double-blind randomized controlled trial. *Archives of Physical Medicine and Rehabilitation*.

[43] Vissers D., Daele U. V., de Hertogh W., de Meulenaere A., Denekens J. (2014). Introducing competency-based education based on the roles that physiotherapists fulfil. *Journal of Novel Physiotherapy and Physical Rehabilitation*.

[44] Lluch Girbés E., Meeus M., Baert I., Nijs J. (2014). Balancing ‘hands-on’ with ‘hands-off’ physical therapy interventions for the treatment of central sensitization pain in osteoarthritis. *Manual Therapy*.

